# Multi-Crop Green LAI Estimation with a New Simple Sentinel-2 LAI Index (SeLI)

**DOI:** 10.3390/s19040904

**Published:** 2019-02-21

**Authors:** Nieves Pasqualotto, Jesús Delegido, Shari Van Wittenberghe, Michele Rinaldi, José Moreno

**Affiliations:** 1 Image Processing Laboratory (IPL), University of Valencia, 46980 Valencia, Spain; jesus.delegido@uv.es (J.D.); shari.wittenberghe@uv.es (S.V.W.); jose.moreno@uv.es (J.M.); 2 Council for Agricultural Research and Economics—Research Centre for Cereal and Industrial Crops, S.S. 673 km 25, 200, 71122 Foggia, Italy; michele.rinaldi@crea.gov.it

**Keywords:** crops, leaf area index, vegetation indices, remote sensing, Sentinel-2, red-edge

## Abstract

The spatial quantification of green leaf area index (LAI_green_), the total green photosynthetically active leaf area per ground area, is a crucial biophysical variable for agroecosystem monitoring. The Sentinel-2 mission is with (1) a temporal resolution lower than a week, (2) a spatial resolution of up to 10 m, and (3) narrow bands in the red and red-edge region, a highly promising mission for agricultural monitoring. The aim of this work is to define an easy implementable LAI_green_ index for the Sentinel-2 mission. Two large and independent multi-crop datasets of in situ collected LAI_green_ measurements were used. Commonly used LAI_green_ indices applied on the Sentinel-2 10 m × 10 m pixel resulted in a validation R^2^ lower than 0.6. By calculating all Sentinel-2 band combinations to identify high correlation and physical basis with LAI_green_, the new Sentinel-2 LAI_green_ Index (SeLI) was defined. SeLI is a normalized index that uses the 705 nm and 865 nm centered bands, exploiting the red-edge region for low-saturating absorption sensitivity to photosynthetic vegetation. A R^2^ of 0.708 (root mean squared error (RMSE) = 0.67) and a R^2^ of 0.732 (RMSE = 0.69) were obtained with a linear fitting for the calibration and validation datasets, respectively, outperforming established indices. Sentinel-2 LAI_green_ maps are presented.

## 1. Introduction

Leaf area index (LAI), or the total one-sided leaf area per unit of ground area (m^2^ leaf per m^2^ surface or dimensionless), can be distinguished in two types. On the one hand, there is the green leaf area index (LAI_green_), representing the leaves which are photosynthetically active, being the most common type of LAI [1], and, on the other hand, there is the brown leaf area index (LAI_brown_), representing the leaf area normalized which is senescent and losing photosynthetic function [2]. The Sentinel-2 mission from the European Space Agency (ESA) has, with the improved optical sensor bands in the red-edge, an increased sensitivity towards LAI_green_ [2], while the shortwave infrared bands are sensitive to cellulose and lignin (dry matter) absorption [2]. Such improved capabilities to obtain more accurate quantifications of LAI_green_ over large areas provides an important aspect in climatic [3], ecological [4] and biogeochemical [5] cycles models, as well as for estimating crop vegetation status [6], developing soil maps [7] and estimating light-use efficiency [8]. Its determination is crucial for the understanding of biophysical processes of crop canopies, being the main morphological parameter used for determining crop growth through the correlation with crop productivity [1,9,10]. In the context of agricultural monitoring, there is a strong interest in estimate LAI_green_ parameter. Near real-time LAI_green_ estimates provides the tool for farmers to obtain the crop health and growth status, further improving the effective technical support in farming practices such as fertilizer application and water management. In this way, increased crop yields and reduced costs and input resources for the agricultural sector are envisaged [11,12]. Remote sensing from satellite, aerial and unmanned aerial vehicle platforms has become a popular technique in monitoring crop LAI_green_ because of its ability to acquire synoptic information at different times and spatial scales [13,14,15]. For agricultural monitoring by remote sensing, the spatial resolution should be at least 20 m and, preferably, 10 m in order to make site-specific management possible [16]. A temporal resolution of less than a week would be required to follow-up acute changes in crop condition and provide timely response in management practices. These requirements are fulfilled by the ESA’s Sentinel-2 mission, providing 10 m pixel size products with a 10-day temporal resolution. Sentinel-2 is a polar-orbiting, superspectral high-resolution imaging mission with twin polar-orbiting satellites, Sentinel-2A and 2B. The mission’s main objective is providing quality information for agricultural and forestry practices and, hence, helping manage food security [17]. With Sentinel-2A in orbit (launched 23 June 2015), the temporal resolution was not yet sufficient for real applications at the individual farmer’s level. But with the additional availability of data from Sentinel-2B (launched 7 March 2017) the revisit period goes down to five days under cloud-free conditions.

LAI_green_ is functionally linked to the canopy spectral reflectance, so its retrieval from optical remote-sensing data has prompted many studies using various techniques [18,19]. Essentially, these retrieval techniques can be classified into two groups, i.e., (1) empirical retrieval methods, which typically consist of relating the biophysical parameter of interest against spectral data through linear (e.g., vegetation indices) or nonlinear (e.g., machine learning approaches) regression techniques [20,21,22,23] and (2) physically-based retrieval methods, which refers to inversion of radiative transfer models (RTMs) against remote sensing observations [24,25,26]. Concerning physical models, experimental studies using RTMs have shown great flexibility in retrieving plant cover variables, because of being able to parameterize these models to a wide range of land cover situations and sensor configurations [27,28]. However, two main drawbacks limit the use of the inversion of RTMs for operational applications. First, RTM approaches typically require some ancillary information to enable the parameterization of the physical model, which may not always be available [13,29]. An additional problem hereby is that if uncertainties are introduced the likelihood increases that the model inversion will not lead to a unique solution and extra steps are required to overcome the ill-posed problem [30]. Second, regardless of the availability of auxiliary data, there is the intrinsic risk of oversimplifying the architecture of canopy for those RTMs fast enough for operational applications. The difficulty in describing canopy structure increases in heterogeneous scenes, such as mosaics of crops at different phenological stages or complex mixtures of woodlands and/or grasslands [2,31,32]. Non-linear regression techniques are standardly used for operational LAI products. For Sentinel-2 an operational LAI product, associated with a quality indicator, is provided through the SNAP (Sentinel Application Platform) toolbox and produced through a neural network which has been trained by simulated spectra generated from well-known RTMs [33]. The algorithm is trained with simulated LAI_green_ values generated from the SAIL radiative transfer model [34], which describes the canopy as a homogenous and horizontal turbid-medium, and the PROSPECT radiative transfer model [35], which considers the leaf as a succession of absorption layers. However, the accuracy of this product is shown improvable [36]. Other machine learning algorithms than neural networks have been proposed to study the retrieval opportunities of LAI from Sentinel-2 and -3 [37], solving the black box problem. However, although machine learning approaches can be fast and can capture the non-linear relationship between different parameters, they are time variant and location dependent [38].

Alternatively, linear empirical models, i.e., vegetation indices (VIs), are one of the most straightforward implementable method in an operational data processing chain. These indices relate a few spectral bands with the biophysical parameter of interest [39] in a way that enhances the spectral characteristics of a given vegetation property while minimizing the soil, atmospheric, and sun-target-sensor geometry effects [22]. Despite the positive aspects of VIs developed for LAI retrieval, their major weakness is the lack of a generally applicable index for multiple vegetation types. The best way to find efficient and robust indices is to use large and diverse field datasets, with a large variety of canopy structures [22,40]. Early studies identified the red and near-infrared (NIR) regions as sensitive to LAI_green_, resulting in the common use of the reflectance broad-bands in these regions through simple ratios [41] or normalized difference ratios [42,43]. It should be mentioned that while these indices were found to be sensitive to low LAI_green_ values_,_ they usually lose sensitivity as LAI_green_ increases (typically above 2–3 according to Haboudane et al. [19]). This saturation of the reflectance at moderate to high LAI_green_ values in the red range (600–700 nm) is due to the high chlorophyll absorption in this spectral range [9]. The wavelength region located in the visible–near infrared (VIS-NIR) transition, i.e., between 690 and 750 nm, generally referred as the “red-edge”, is the region between maximum chlorophyll absorption in the red, and maximum reflection (high scattering) in the NIR caused by leaf cellular structure abundance, i.e., LAI [44,45,46]. Reflectance in the red-edge transition region is much higher than in the visible range especially for these moderate to high LAI_green_ values, where the upwelling radiance in the red-edge range provides a higher and less noisy signal compared to the low values in the red region. It has been specifically demonstrated, through real [47,48] and simulated spectral data [42,46], that the shape of the red-edge region and mainly the slope is strongly influenced by chlorophyll density and, hence, by LAI_green_. Despite this well-known sensitivity, practically no established indices use the red-edge region for the LAI_green_ retrieval as until now no free operational satellites had narrow-bands in this region. With the Sentinel-2 satellites (13 spectral bands) not only optimal and temporal resolution for crop monitoring is guaranteed, but, moreover, also spectral configuration in the red-edge is improved, with narrow-bands centred at 705 nm (B5) and 740 nm (B6). Recent studies have explored the potential of Sentinel-2 for the LAI_green_ retrieval based on simulated datasets [49,50]. But at this moment, few studies have used real Sentinel-2 images in combination with in situ datasets for agricultural applications. Moreover, these studies using the red-edge Sentinel-2 bands for LAI_green_ retrieval, calibrated and validated their products for only a few crop types [51,52], leaving the robustness of a generic retrieval application still an open issue.

In this respect, we aim to develop a simple, accurate empirical algorithm for deriving LAI_green_ from Sentinel-2 real data of multi-crop agricultural fields, using two large in situ field datasets. The first objective is to determine if the commonly used VIs for estimating LAI_green_ may be applicable for a variety of crop types. Secondly, we want to identify the Sentinel-2 spectral bands that present the highest correlation for the estimation of a wide variation in crop LAI_green_. Based on this analysis and on a parallel study of the importance of the new Sentinel-2 red-edge bands, a new robust LAI_green_ index is defined. The performance of the new index and established VIs indices are validated and applied over two distinct agricultural test sites.

## 2. Materials and Methods 

### 2.1. Study Sites

**Figure 1:**
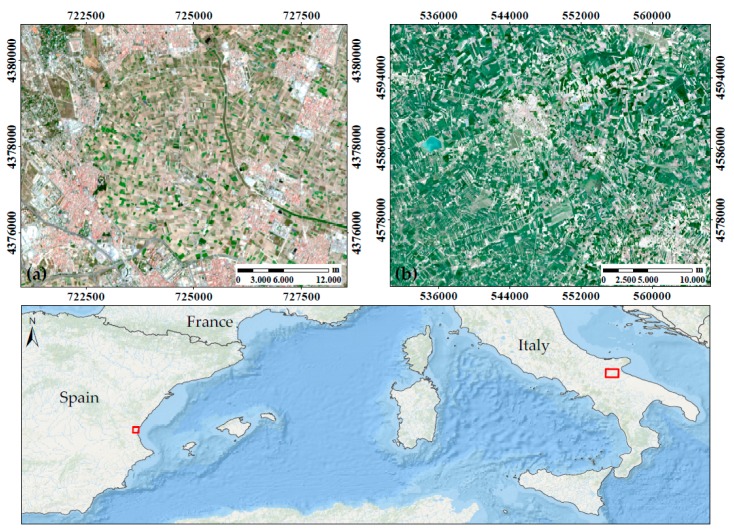
Test site locations. **(a)** Valencia (Spain) from the Sentinel-2 image of 6 April 2017, UTM-WGS84 Zone 30S **(b)** Foggia (Italy) from the Sentinel-2 image of 8 April 2017, UTM-WGS84 Zone 33T.

Field data was collected at two study sites in the Mediterranean region (Figure 1). The first site located in Valencia (Spain), named Huerta of Valencia, is an area in an alluvial plain between the Turia river, the Mediterranean Sea and Albufera lake, with an approximate area of 12,000 hectares. The climate is typically Mediterranean with mild, wet winters and hot dry summers, and a yearly average temperature around 18 °C. Seasonal rainfall is minimal in summer and maximal in autumn and spring, with an average annual value of 454 mm [53]. A complex historical irrigation system based on irrigation ditches brings water to this fertile soil in which cereals, vineyards and olive trees were originally the main crops and nowadays accompanied by rice, tigernut and new species of vegetables and citric orchards. All these crop types are currently cultivated in small plots of size 40–100 m. For this study, crop fields were measured in the Burjassot, Moncada and Alboraya municipalities (study site central coordinates 39°31’11.73’’ N, 0°23’20.48’’ W, 18 m above sea level, Datum WGS84).

The second test site is an Italian agricultural area located near Foggia (study site central coordinates 41°27’36.76’’ N, 15°32’45.33’’ E, 70 m a.s.l., Datum WGS84). The climate is Mediterranean, but with a marked continental influence being 30 km distant from the coast. This promotes abrupt seasonal and daily temperature changes, sometimes as high as 20 °C. The average annual temperature is 15.8 °C but summers can be very hot and dry, with temperatures easily exceeding 35 °C, and winters with temperatures close to 0 °C. Rainfall is usually between 350 mm and 700 mm (average 469 mm), occurring mainly during autumn and winter [54]. The agricultural sector is the mainstay of Foggia’s economy, where grapefruit, olives, durum wheat and tomato are the majority crops for centuries. The industries present are mostly devoted to food processing, with tomato processing the major industry branch.

### 2.2. Green Leaf Area Index (LAI_green_) Datasets

**Figure 2:**
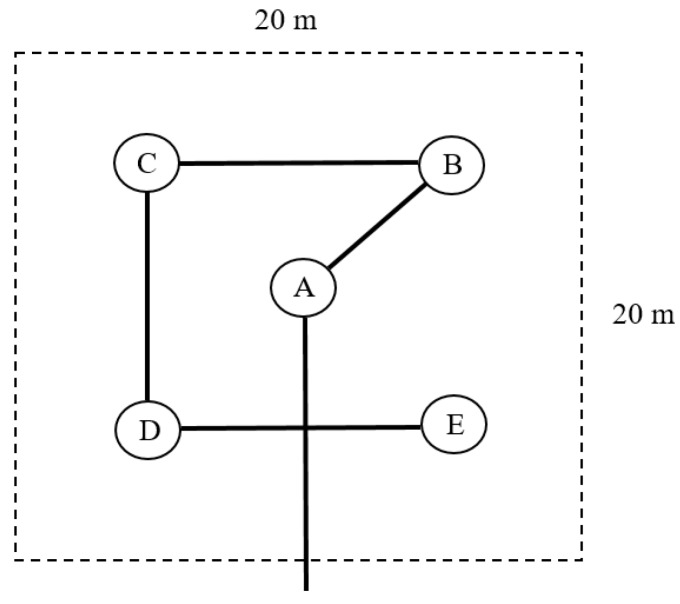
Sampling approach for each elementary sampling unit (ESU).

At each study site, a large LAI_green_ dataset was collected with the LAI-2200 Plant Canopy Analyzer [55], which uses a fish-eyes lens with a hemispheric field of view (±45°). The detector is composed of five concentric rings (sensitive to radiation below 490 nm). Each ring responds over a different range of zenith angles and radiation is, thus, azimuthally integrated. The measurements were collected in one sensor mode using a 180° view cap, in clear sky condition, to avoid interferences from a user’s shadow, following the Land European Remote-Sensing Instruments (VALERI) field protocol (http://w3.avignon.inra.fr/valeri/). The VALERI protocol is a sampling strategy corresponding to high spatial-resolution satellite imagery, choosing elementary sampling units (ESUs) of 20 m × 20 m for each measuring plot. Each ESU was chosen in the middle of the crop field which had minimum dimensions of 40 m × 40 m in Valencia and 100 m × 100 m in Foggia. A minimum distance of 20 m from the edges of the field was kept. To account for the spatial LAI_green_ variability within each ESU, measuring points were sampled following a square spatial sampling with 5 measurements at each point (A, B, C, D and E), providing a statistically mean LAI_green_ estimate per ESU (Figure 2). The centre of the ESU (sampling point A) was geo-located using a GPS providing an accuracy of less than 5 m for later matching the mean LAI_green_ estimate with the corresponding Sentinel-2 reflectance data. The field protocol was identic for both study sites.

**Figure 3:**
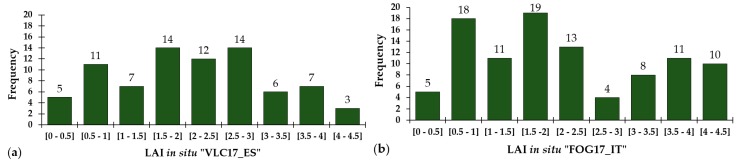
ESUs frequency histogram classified for LAI_green_ value range, for (**a**) the VLC17_ES testing dataset and (**b**) the FOG17_IT validation dataset. Bare soil ESUs are not included.

The field dataset collected in Valencia (Figure 1a), hereafter called “VLC17_ES”, consists of 79 average LAI_green_ values for the respective ESUs sampled on 22 and 23 May; 18 and 19 July; and 8 and 9 November 2017. Several dates throughout the season were selected to cover a wider variety of growth stages. In total, LAI_green_ data from 79 ESUs were taken containing orange tree (*Citrus x sinensis*), collard (*Brassica oleracea*), tigernut (*Cyperus esculentus*), potato (*Solanum tuberosum*), artichoke (*Cynara scolymus*), squash (*Cucurbita pepo*), alfalfa (*Medicago sativa*), broad bean (*Vicia faba*), watermelon (*Citrullus lanatus*), pumpkin (*Cucurbita maxima*), onion (*Allium cepa*), celery (*Apium graveolens*) and lettuce (*Lactuca sativa*). The number of ESUs, classified by LAI_green_ value range, is given in the corresponding histogram of the VLC17_ES dataset (Figure 3a). Furthermore, 13 bare soil ESUs were included (LAI_green_ = 0), with the aim to create a more robust and general method.

The field dataset collected in Foggia (Figure 1b), hereafter called “FOG17_IT”, was taken in the framework of the H2020 SENSAGRI (Sentinels Synergy for Agriculture, http://sensagri.eu/) project. This dataset consists of 99 average LAI_green_ values collected on the 16, 21, 22 and 29 March; 5 and 13 April; 11, 17 and 30 May; 12, 15 and 21 June 2017. Mean LAI_green_ data of durum wheat (*Triticum durum*), tomato (*Solanum lycopersicum*) and horse bean (*Vicia faba*) ESUs were measured, with the number of ESU’s specified in the histogram of Foggia test site (Figure 3b). In addition, 10 bare soil ESUs were included.

The two standard and independently collected field datasets VLC17_ES (n = 79) and FOG17_IT (n = 99) were, respectively, used as testing and validation dataset for the LAI_green_ index testing and development. Both datasets are covering a wide range of crop LAI values, i.e., from 0 to 4.5, providing an optimal experimental dataset for the definition of a new general methodology.

### 2.3. Sentinel-2 Imagery and Sentinel Application Platform (SNAP) LAI Product

All field campaigns were carried out on days close, with a maximum of five days’ difference, to overpass dates of Sentinel-2 over the study area. Each Sentinel-2 satellite carries a Multi-Spectral Imager (MSI) instrument/sensor with a swath of 290 km on board. The MSI provides a versatile set of 13 spectral bands spanning from the visible and NIR to the shortwave infrared (SWIR) (443–2190 nm), featuring four bands at 10 m (VIS and NIR bands), six bands at 20 m (red-edge and SWIR) and three bands at 60 m spatial resolution for atmospheric correction (Table 1).

The images were downloaded directly and free of charge from the ESA server (https://scihub.copernicus.eu/). ESA provides Level-1C images, being geometrically corrected [56], with top-of-atmosphere (TOA) reflectance; and Level-2A images, being geometrically and atmospherically corrected, with top-of-canopy (TOC) reflectance. We downloaded 11 available cloud-free Level-1C acquisitions of Sentinel-2 over Valencia and Foggia study areas (Table 2). In addition, we used the SNAP toolbox to process these Level-1C images into Level-2A data, with the retrieval of the LAI_green_ product accompanied by a product quality indicator [33].

For each ESU the TOA reflectance spectrum was obtained from the central pixel of the corresponding plot of the Sentinel-2 image. These images were atmospherically corrected using the Sen2Cor procedure available in the Sentinel-2 SNAP toolbox, converting TOA reflectance into TOC reflectance [57]. The Sentinel images were resampled to 10 m pixel size with all selected pixels falling entirely inside the corresponding ESU. Subsetting was done to reduce the image size and the processing time, and to cover only the study areas.

### 2.4. Established Vegetation Indices Analysis

The performance of commonly used LAI_green_ indices was tested (Table 3, indices shaded), with the specific bands as defined by the original authors. The accuracies of each index was specifically analyzed with linear (f(x) = ax + b), polynomial of second order (f(x) = ax^2^ + bx + c), exponential (f(x) = a × exp(bx)) and exponential of second order fitting (f(x) = a × exp(bx) + c × exp(dx)). Additionally, we introduced several generic index formulations, i.e., with undefined specific bands, into a Matlab-based graphical user interface toolbox called ARTMO (Automated Radiative Transfer Models Operator) [58]. ARTMO consists of multiple RTMs and several retrieval toolboxes that enable the development and optimization of retrieval algorithms to convert optical images into maps of vegetation properties. The indices formulation introduced in ARTMO was based on commonly used LAI_green_ indices, among other VIs typically used to estimate various biophysical variables such as chlorophyll (Table 3, non-shaded indices). The spectral indices assessment toolbox [59] was used to calibrate and validate generic indices, i.e., looking for those wavelengths that provide the best correlation with LAI_green_, using the VLC17_ES testing dataset. A cross-validation method with the k-fold technique was used to ensure more robust results [60]. This method divides the available data into k subsets. From these k sub-datasets, k-1 sub-datasets are selected as a calibration dataset and a single k sub-dataset is used for model validation. The cross-validation process is then repeated k times, with each of the k sub-datasets used as validation dataset. Thus, all VLC17_ES field data are used for both calibration and validation. Here, we used a 4-fold (k = 4) cross-validation procedure.

The selection of the index and, accordingly, the best performing bands based on the testing dataset will rely on a series of criteria. First, it should be a simple index, preferably using only two bands to minimize processing time and improve its operational use. Secondly, the index must have physical sense, that is, it should be based on areas of the spectrum with high influence of the LAI_green_ parameter. As mentioned earlier, the red, red-edge and NIR regions are the areas with the greatest influence of this parameter, so the Sentinel-2 bands used by the proposed index will be from these spectral regions. Third, it must present good statistics when applied to the completely independent validation dataset (FOG17_IT). The coefficient of determination (R^2^) and root mean squared error (RMSE) will be selected as indicators of the accuracy of the statistical estimation models.

## 3. Results

In this section we first present the quality results of Sentinel-2 LAI product, comparing them with both the Valencia and Foggia LAI_green_ in situ datasets. In the same way, we show the performance of the common indices used by the bibliography, presenting graphs with the best settings for each index. Next, the best band combination for each commonly used index, obtained with ARTMO toolbox, are shown, defining finally the new SeLI index and showing its validation assessment with the Foggia dataset.

### 3.1. Performance of the Sentinel-2 Level-2A LAI_green_ Product

**Figure 4:**
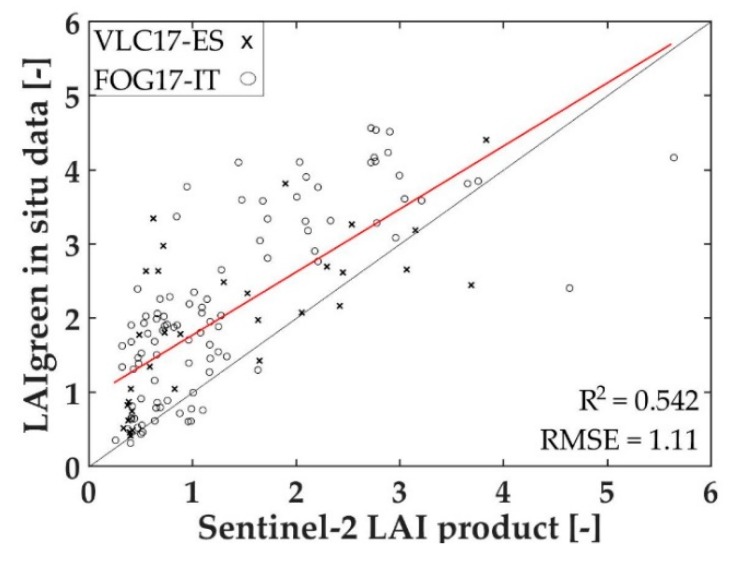
Green leaf area index (LAI_green_) in situ values in comparison to the Sentinel-2 LAI_green_ product, with 1:1 line (black), its statistics and linear fitting (red).

The LAI_green_ products of both Valencia and Foggia Sentinel-2 images were obtained and compared with the in situ LAI_green_ of the corresponding pixel. The analysis was carried out with only 31 LAI_green_ VLC17_ES values and 99 FOG17_ES values, because only those corresponding pixels indicated with a good product quality flag were selected. Figure 4 shows the Sentinel-2 LAI_green_ product values and the in situ LAI_green_ values, with the 1:1 line (black), its statistics and the linear fitting (red). A clear underestimation of the product values is shown in Figure 4.

### 3.2. Performance of Common LAI_green_ Indices for a Multi-Crop Dataset

Commonly used LAI_green_ indices (Table 3, indices shaded) were evaluated with their default bands using the multi-crop VLC17_ES dataset. The R^2^ obtained with different types of fitting functions ranged between 0.234–0.663 when applying the respective indices on the multi-crop dataset (Table 4). Hence, the accuracies of each index obtained with linear, polynomial of second order, exponential and exponential of second order fitting, were rather low. The p-value is <0.001 in all cases except for the ratio index (RI).

**Figure 5:**
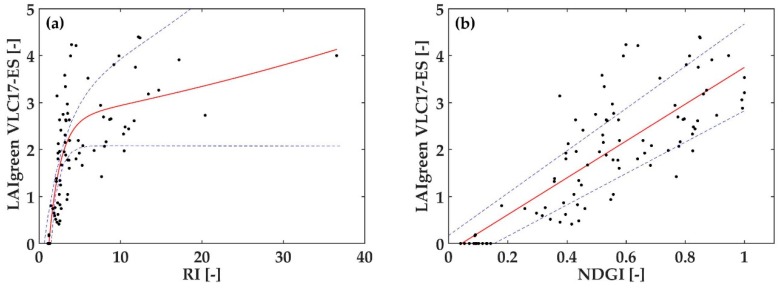

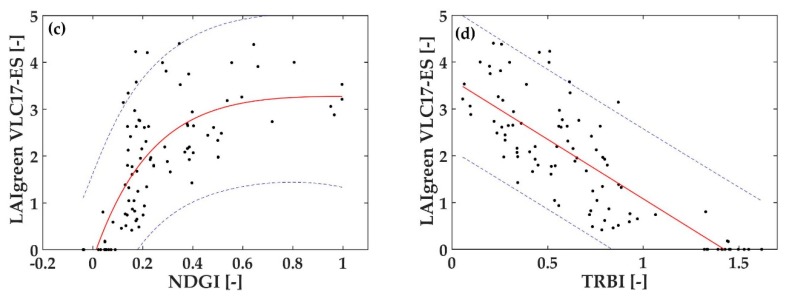
Graphic representation of the LAI_green_ of VLC17_ES dataset as a function of the established LAI_green_ indices, with the best fitting function and 95% of confidence interval. (**a**) RI [41]; (**b**) NDGI [43]; (**c**) NDGI [42]; (**d**) TRBI [61].

Each established index was represented as a function of the VLC17_ES LAI_green_ values, to show its predicting performance (Figure 5). As can be seen, they generally present a scattered performance and all indices present a saturation problem with high and/or low LAI_green_ values. Concretely, the RI index (Figure 5a) overestimates to an extreme extent at low and high LAI_green_ values. The TRBI (Figure 5d) shows a saturation process with LAI_green_ values close to 3. And both normalized indices (Figure 5b,c) already present their greater value (the unit), with also LAI_green_ values close to 3. The normalized index defined by Delegido et al. (2011) uses a red-edge band (705 nm), but as it is used in combination with the red band (665 nm) [42], the saturation problem at high LAI_green_ values appears. So, the effectiveness of LAI_green_ indices depends entirely on the combination of bands used.

**Figure 6:**
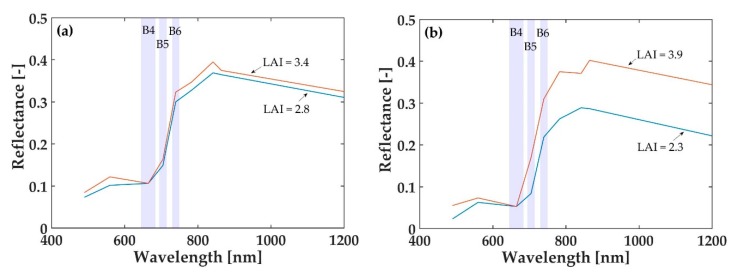
Representation of real Sentinel-2 top-of-canopy (TOC) reflectance spectra of **(a)** artichoke crops (20/07/2017 Sentinel-2 image used) and (**b**) orange tree crops (07/11/2017 Sentinel-2 image used). The red and red-edge Sentinel-2 bands (B4, B5 and B6) are represented with their corresponding bandwidth.

Another demonstration of the saturation produced by the band located at the red region (B4: 665 nm) is Figure 6. In this Figure, real Sentinel-2 spectra of artichoke (LAI = 2.8 in blue, 3.4 in orange) and orange tree crops (LAI = 2.3 in blue, 3.9 in orange) with moderate-high LAI_green_ values of the VLC17_ES dataset are represented. Values higher than 2 have been chosen because it is usually the limit at which saturation process starts with common LAI_green_ indices (Figure 5). As can be seen, they have the same reflectance value in the 665 nm band, that is, at moderate-high LAI_green_ values, chlorophyll maximally absorbs in this region, producing saturation. At the same time, the red-edge band close to the red (B5: 705 nm) does not show such entire absorption saturation, which brings the advantage of using red-edge Sentinel-2 bands for LAI_green_ estimation.

### 3.3. Sensitivity of Spectral Bands against LAI_green_ Parameter

In order to investigate more generic index options, the following step involved systematically calculating all band combinations with the ARTMO toolbox. Table 5 lists the 10 best statistical results obtained for each generic index and the corresponding bands with a linear fit, using the VLC17_ES dataset and a cross-validation process. The analysis was performed with linear fitting, because the aim of this study is to define a simple relationship between the LAI_green_ parameter and Sentinel-2 bands, analysing whether there is a linear relationship between the LAI_green_ in situ values and the estimated values. Furthermore, as seen in Table 4, linear fitting produces one of the best statistical results.

Comparing to the tested established indices, these results already show a more promising correlation with a R^2^ ranging between 0.701 and 0.737. However, questions arose when evaluating the obtained wavelengths of the resulting best-performing bands from a physical point of view. In the majority of cases, the selected bands were physically not only influenced by chlorophyll absorption, but mainly by other leaf constituents such as lignin, cellulose and water (e.g., 1610 nm, 2190 nm) affecting the scattering properties in the NIR and SWIR [69,70]. The only case where physical chlorophyll-related bands were chosen, was the NDGI, using one of the new red-edge bands (705 nm, in the tail of the chlorophyll absorption peak) and the other in the NIR region (865 nm). The red band (B4) appeared not to be chosen at all.

### 3.4. Optimized Simple Index for LAI_green_ Retrieval from Sentinel-2 Data: SeLI

According to the previous outcome of the NDGI, we used this index structure and analysed all band combinations in red, red-edge and NIR regions, i.e., the bands 4, 5, 6, 7, 8 and 8a (Table 1). Table 6 summarizes the normalized difference ratio index band combinations with their corresponding statistics for the independent testing and validation datasets. The statistical results are ranked according their performance for the testing dataset. All indices perform better compared to the common used indices, apart from those listed at the bottom. The top four performing combinations do not use the red band, but instead, all use the red-edge band at 705 nm in combination with a far-red or NIR band.

**Figure 7:**
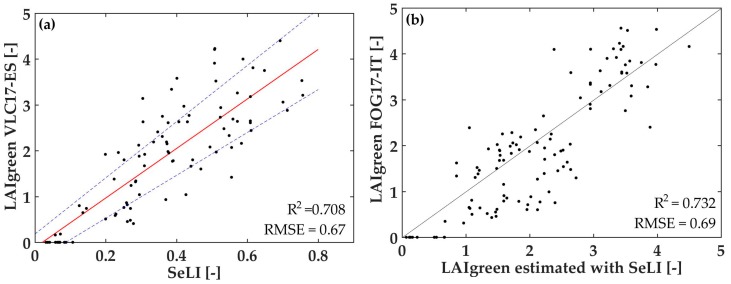
(**a**) Graphic representation of the LAI_green_ of the testing VLC17_ES dataset (n = 92) as a function of SeLI, the index proposed, with linear fit and 95% of confidence interval, (**b**) LAI_green_ in situ validation data from FOG17_IT (n = 109) as a function of LAI_green_ estimated with SeLI, with 1:1 line and its statistics.

As expected, the best result for the testing dataset is again the 705 (B5)–865 (B8a) nm combination (R^2^ = 0.708), and, moreover, confirmed by the validation dataset (R^2^ = 0.732). These both independent datasets on real in situ data give us the strong experimental proof that the LAI_green_ parameter presents a linear behavior up to values of five, proposing therefore a physiologically-based LAI_green_ index, hereafter called the Sentinel-2 LAI_green_ Index (SeLI) (Equation (1)). For the in situ VLC17_ES LAI_green_ dataset (Figure 7a), it is observed that SeLI values vary from 0.03, corresponding to bare soils, to a maximum of 0.76, corresponding to LAI_green_ values up to 4.5, with potato and alfalfa crops showing the highest LAI_green_ values. We do not observe any saturation at these high LAI_green_ values, while previously shown indices did. Hence, SeLI showed the potential to be used in a unified algorithm for LAI_green_ estimation in different crop types.

(1) $$\mathrm{SeLI}\;=\frac{R_{865\;}-\;R_{705}}{R_{865}\;+\;R_{705}}$$

From Figure 7a, the LAI_green_ estimation equation through SeLI index is extracted, with a linear fit (Equation (2)). We have tested, besides the linear fitting (R^2^ = 0.708, RMSE = 0.67), also an exponential fitting (R^2^ = 0.603, RMSE = 0.78) and a polynomial of second order (R^2^ = 0.727, RMSE = 0.65) to fit the index with in situ LAI values. The polynomial fitting presents slightly higher statistics, but the adjustment is negative, not presenting much physical sense.

*LAI_green_* = 5.405 × *SeLI* − 0.114 (2)

Figure 7b shows the LAI_green_ values estimated with Equation (2) together to the validation dataset taken in Foggia, obtaining a correlation R^2^ of 0.732 and RMSE of 0.69 (1:1 line), showing strong validation statistics.

**Figure 8:**
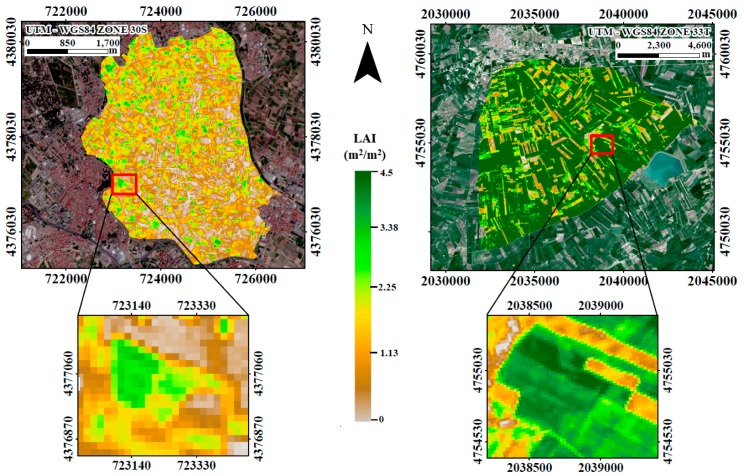
LAI_green_ maps estimated with SeLI proposed index in Valencia test site using the Sentinel-2 image of 26 May 2017 (left) and Foggia test site using the Sentinel-2 image of 8 April 2017 (right). Note the different scale of both study sites and their respective agricultural areas.

Finally, we applied the SeLI to both field sites, using the 26 May 2017 and 8 April 2017 Sentinel-2 images for the Huerta of Valencia and Foggia site, respectively. The resulting maps are shown in Figure 8, demonstrating the applicability of SeLI at high spatial resolution for two distinct agricultural areas. In brown, the pixels with the lowest value of LAI_green_ are shown, mainly corresponding to bare soils, and in green colour, the pixels with the highest LAI_green_ value, corresponding to potato and alfalfa crops in the case of Valencia, and wheat in the case of Foggia. A zoom of each map is shown to demonstrate the high spatial resolution of Sentinel-2, being able to observe individual agricultural plots of 40 m × 40 m (Valencia) and 100 m × 100 m (Foggia) based on LAI, while also observing slight LAI variability within these plots.

## 4. Discussion

With the availability of a narrow band in the red-edge region by Sentinel-2, an improved and simple estimation of LAI_green_ based on a simple index becomes possible at high spatial resolution. The proposed SeLI index shows a significant improvement towards indices using the saturating bands in the red (B4 in Sentinel-2). Moreover, no saturation appeared in the obtained LAI_green_ product based on the red-edge bands (B5: 705 nm and B6: 740 nm). The B4 (665 nm) saturation at high LAI_green_ values is clearly shown with real Sentinel-2 TOC reflectance spectra for different LAI_green_ values, higher than 2 (Figure 5 and Figure 6). The red-edge bands (B5: 705 nm and B6: 740 nm) in contrast are both affected by higher scattering, whereby the B5 band is still driven by chlorophyll absorption. This agrees with numerous authors who emphasize the importance of the red-edge bands for the estimation of biophysical parameters, mainly the LAI_green_ and chlorophyll estimation [42,46,71]. The proposed Sentinel-2 LAI Index (SeLI) exploits the B5 red-edge band, which has been widely demonstrated that is highly influenced by the LAI_green_ parameter [46,47,48], and the B8a NIR band, which is driven by the scattering changes in moderate-to-high LAI values in crops [72]. Very few previous indices have used bands in the red-edge region because no free operational previous sensors had narrow bands in this spectral area [42]. Both linear and non-linear empirical regression techniques have been tested for the LAI retrieval on simulated spectrally resampled airborne data [50,73] and recently on real Sentinel-2 data [10,52]. The band selection obtained from these methodologies appeared to favor (1) green and SWIR bands in the case of linear regression by VIs, and (2) red, NIR and SWIR bands in the case of non-linear regression by machine learning approaches [74]. These bands were also chosen by several of our tested VIs (Table 5), with the difference that the NDGI formulation indicated the use of red-edge (705 nm) and a NIR band (865 nm) as best band selection.

The robustness and generality of the SeLI index is demonstrated by applying it to an independent in situ field dataset from a distinct geographical location with crop types different from those included in the testing dataset, obtaining equally good statistics (R^2^ of 0.732, RMSE of 0.69). Specifically, SeLI does not present problems of saturation when it is applied to a multi-crop Valencia in situ dataset composed of 13 different crop types and LAI_green_ values that go up to 4.5, obtaining R^2^ of 0.708 and RMSE of 0.67. Furthermore, when SeLI is applied to the Foggia region, characterized by high LAI_green_ values, the limits of the different crop fields and LAI_green_ variability within the crop field appears even for the high value ranges, indicating variable growing conditions. Such a clear distinction in LAI_green_ variability allows evaluation of management practices at the field level. Hence, it is shown that the SeLI index generally can be applied for LAI_green_ retrieval of different crop types and distinct areas. A limitation of the index is that it has been calibrated and validated with LAI data up to 5, so it is only applicable to agricultural areas with this range of values, although it is the common range of in situ LAI measured values in a lot of studies with a great variety of crop types; such as wheat [75,76], corn [19], potato [10] and sugar beet [2,47]. Currently, ongoing scientific debate is taking place on the discussion if there is a linear relationship or not between in situ LAI values and estimated values [77]. Our study shows that in situ data are linearly related to SeLI, in the value range of 0 to 5. This result is in accordance with other results in which also LAI of agricultural areas is estimated through indices and linear models in similar ranges [10,75,78,79,80]. Generally, no values higher than 5 are used in these studies constraining the model applicability to this range. As SeLI has a physiological foundation, the index will be applicable to a higher value range, but the SeLI-LAI fitting relationship might change depending on the dataset range. To verify this, a LAI in situ data range >5 would be required. However, one must also consider the instrumental limitations for in situ measurements. The LAI-2200 Plant Canopy Analyzer [55] calculates LAI by comparing differential light measurements above and below canopy. The maximum measurable LAI is generally lower for these devices measuring gap fraction with LAI reaching an asymptotic saturation level at a value of about 5, compared to that assessed via destructive methods. The cause for this is gap fraction saturation as LAI approaches five or six [81,82,83].

In this work, the Sentinel-2 LAI_green_ product obtained from the SNAP toolbox was also tested with the multi-crop dataset from Valencia. The results show underestimated LAI estimations (R^2^ = 0.475, RMSE = 0.91). There are some studies, which have also compared this Sentinel-2 LAI_green_ product with in situ LAI_green_ crop data, that obtain better R^2^ [84,85], but they only tested the product with few crop types. When the product is analysed in different areas and plant species, the results can be improved [36]. This finding could be explained by the fact that the Sentinel-2 algorithm used for land surface parameters, including LAI_green_ product, ingests almost all spectral bands and applies a nonlinear regression to estimate each parameter [33], in addition to the fact that it has been proven that there is a substantial sensitivity of Sentinel-2 biophysical products to the implemented rugged terrain corrections [36].

The other main challenge in the retrieval of biophysical parameters with vegetation indices is the difficulty of finding a simple index with such a general character that it can estimate the parameter of a wide variety of crop types. In this work it has also been shown that the established indices do not present this general character. This may be because they were developed and calibrated based on limited experimental data in terms of species, presenting improvable statistics (R^2^ between 0.234–0.663) when applied to multi-crop datasets. In an attempt to improve estimations over this multi-crop dataset, all band combinations were systematically calculated for each index in order to achieve the highest possible correlation for the estimation of LAI_green_. More promising results were obtained, with a R^2^ between 0.701 and 0.737. However, when inspecting these sensitive bands whether they are physically meaningful, i.e., if the selected bands are actually influenced only or mostly by LAI_green_, then these indices turned out to be questionable. In the majority of cases, the selected bands were influenced by leaf constituents such as lignin, cellulose and water (e.g., 1610 nm, 2190 nm) affecting the scattering properties in the NIR and SWIR [69,70], and being less related to photosynthetically based LAI_green_. At the field or landscape scale, canopy reflectance patterns represent the integrated effects of all biophysical parameters. Co-variation mechanisms of leaf constituents is typically causing the selection of bands related to other covarying biochemicals such as pigments or lignin due to their high effect on spectral variability [86]. Similarly, it was earlier observed that due to the covariation between water content and chlorophyll content (related with LAI parameter), typically bands in the water content absorption region are selected as most sensitive [87]. To improve the estimation of LAI_green_ (aside from LAI_brown_), bands only affected by structural leaf components should be omitted. Structurally-related NIR and SWIR bands may improve the LAI_green_ retrieval when the model is trained on healthy vegetation [74] but may be less generally applicable for scenarios with different structural types or stress conditions. With the band selection B5 and B8a, SeLI is functionally related to green LAI, avoiding absorption saturation in the red region.

It should be mentioned that this is the first time that this kind of LAI_green_ retrieval can be carried out for agricultural areas with plots sizes of only 40–100 m, such as Huerta of Valencia, due to the lack of an operational satellite with the required spatial and temporal resolution. ESA’s satellite Sentinel-2 aims to replace and improve the older generation of satellite sensors such as Landsat and SPOT, with improved spectral and spatial capabilities. Therefore, the Sentinel-2 satellites provide a great opportunity for global vegetation monitoring, and specifically crop field monitoring, due to its enhanced spatial, spectral and temporal characteristics [42].

Finally, further validation is required with other field campaigns and synthetic Sentinel-2 data to reinforce findings. Considering appropriate instrumental tools, the index behavior for LAI_green_ values higher than 5 should be tested, as well as the fitting behavior of these further ranges.

## 5. Conclusions

Numerous VIs have been proposed for the estimation of green leaf area index (LAI_green_) over various crop types, but the general problem appears when they are applied to multi-crop datasets, obtaining low estimation accuracies and additionally at LAI_green_ values higher than 2, saturation problems appear. Based on the availability of Sentinel-2 narrow red-edge bands, we explored new index possibilities for accurate LAI_green_ estimations for heterogeneous agricultural areas, based on spectral areas influenced mainly by photosynthesis-related absorption regions. The proposed Sentinel-2 LAI Index (SeLI) is a normalized index that uses the new Sentinel-2 narrow B5-band located at the beginning of the red-edge region (705 nm), a spectral area which balances the influence of strong chlorophyll absorption and minimal scattering at moderate-high LAI_green_ values, and a NIR band (865 nm) influenced by leaf scattering, as a reference band. SeLI was calibrated with a multi-crop Valencia dataset composed of LAI_green_ values of 13 different crop types, obtaining a R^2^ of 0.708 and RMSE of 0.67. The validation of SeLI proved good, with a R^2^ of 0.732 and RMSE of 0.69. This work demonstrated the existence of a linear relationship between in situ LAI_green_ and the spectral information of Sentinel-2, in the range of 0 to 5. Sentinel-2 generated maps over the Valencia and Foggia test sites convincingly illustrate the great potential of high spatial resolution LAI_green_ monitoring at the single agricultural plot level, even for small- and medium-scale agricultural activities.

## Figures and Tables

**Table 1:** Sentinel-2 bands setting.

Band Number	Function	Central Wavelength (nm)	Bandwidth (nm)	Spatial Resolution (m)
1	Coastal aerosol	443	27	60
2	Blue	490	98	10
3	Green	560	45	10
4	Red	665	38	10
5	Vegetation red-edge	705	19	20
6	Vegetation red-edge	740	18	20
7	Vegetation red-edge	783	28	20
8	Near infrared (NIR)	842	145	10
8a	Vegetation red-edge	865	33	20
9	Water vapour	945	26	60
10	Shortwave infrared (SWIR)-cirrus	1380	75	60
11	SWIR	1610	143	20
12	SWIR	2190	242	20

**Table 2:** Sentinel-2 images used in each field campaign.

Location	Field Work Date (2017 year)	Sentinel-2 Image Code
Valencia	22 and 23 May	S2A_MSIL1C_20170526T105031_N0205_R051_T30SYJ_20170526T105518
	18 and 19 July	S2A_MSIL1C_20170720T105029_N0205_R051_T30SYJ_20170720T105641
	8 and 9 November	S2A_MSIL1C_20171107T105229_N0206_R051_T30SYJ_20171107T131035
Foggia	16 March	S2A_MSIL1C_20170319T095021_N0204_R079_T33TWF_20170319T095021
	21 and 22 March	S2A_MSIL1C_20170319T095021_N0204_R079_T33TWG_20170319T095021
	29 March	S2A_MSIL1C_20170329T095021_N0204_R079_T33TWF_20170329T095024
	5 and 13 April	S2A_MSIL1C_20170408T095031_N0204_R079_T33TWF_20170408T095711
	11 and 17 May	S2A_MSIL1C_20170518T095031_N0205_R079_T33TWF_20170518T095716
	3 May	S2A_MSIL1C_20170528T095031_N0205_R079_T33TWF_20170528T095531
	12 June	S2A_MSIL1C_20170607T095031_N0205_R079_T33TWF_20170607T095031
	15 and 21 June	S2A_MSIL1C_20170617T095031_N0205_R079_T33TWF_20170617T095546

**Table 3:** Generic vegetation indices introduced in Automated Radiative Transfer Models Operator (ARTMO), where indices based on commonly LAI_green_ indices are shown shaded and typically indices used to estimate other biophysical parameters are shown non-shaded. R_λ_ represents reflectance at the wavelength λ. The generic name of each index has been established in this study.

Based Reference	Formula	Generic Name	Abbreviation	Generic Formula
[41]	$\frac{R_{800}}{R_{675}}$	Ratio Index	RI	$\frac{R_{1}}{R_{2}}$
[43]	$\frac{R_{800}-R_{670}}{R_{800}+R_{670}}$	Normalized Difference Generic Index	NDGI	$\frac{R_{1}-R_{2}}{R_{1}+\;R_{2}}$
[42]	$\frac{R_{704}-R_{665}}{R_{704}+R_{665}}$			
[61]	$\frac{R_{520-600\;}+R_{630-690}}{R_{760-900}}$	Three Ratio Band Index	TRBI	$\frac{R_{1}+R_{2}}{R_{3}}$
[62]	$\frac{R_{680\;}-R_{500}}{R_{750}}$	Three Difference Band Index	TDBI	$\frac{R_{1}-R_{2}}{R_{3}}$
[63]	$\frac{R_{750}-R_{710}}{R_{710}-R_{680}}$	MERIS Terrestrial Generic Index	MTGI	$\frac{R_{1}-R_{2}}{R_{2}-R_{3}}$
		Normalized Difference 3 band	ND3b	$\frac{R_{1}-R_{2}}{R_{2}+R_{3}}$
[64]	$\frac{R_{750}-R_{705}}{R_{750}+R_{705}-2R_{445}}$	Multi-band Normalized Index	MNI	$\frac{R_{1}-R_{2}}{R_{1}+R_{2}-R_{3}}$
[65]	$\frac{R_{550}-R_{670}}{R_{550}+R_{670}-R_{480}}$			
[66]	$R_{676}-0.5\left(R_{746}+R_{665}\right)$	Generic Line Height	GLH	$R_{1}-0.5\left(R_{2}+R_{3}\right)$
[67]	$0.5[120(R_{750}-R_{550})-200\left(R_{670}-R_{550}\right)$]	Triangular Generic Index	TGI	$0.5[120(R_{1}-R_{2})-200\left(R_{3}-R_{2}\right)$]
[68]	$[(R_{700}-R_{670})-0.2\left(R_{700}-R_{550}\right)]\left(R_{700}/R_{670}\right)$	Modified Chlorophyll Generic Index	MCGI	$[(R_{1}-R_{2})-0.2\left(R_{1}-R_{3}\right)]\left(R_{1}/R_{2}\right)$

**Table 4:** Statistics obtained with a linear, polynomial of second order, exponential and exponential of second order fitting for each index. The best fitting is boldfaced.

Index	References	Linear Fitting		Polynomial Fitting, Second Order		Exponential Fitting		Exponential Fitting, Second Order	
		R^2^	RMSE	R^2^	RMSE	R^2^	RMSE	R^2^	RMSE
RI	[41]	0.355	0.91	0.314	0.93	0.234	1.13	**0.663**	0.75
		y = 0.15x + 1.11		y = −0.01x^2^ + 0.33x + 0.55		y = 1.51exp(0.04x)		y = 2.59exp(0.01x) − 6.19exp(-0.71x)	
NDGI	[43]	**0.659**	0.72	0.612	0.74	0.571	0.85	0.629	0.79
		y = 3.93x − 0.18		y = −1.98x^2^ + 5.93x − 0.55		y = 0.68exp(1.78x)		y = − 1547exp(3.96x) + 1548exp(3.96x)	
	[42]	0.402	0.86	0.389	0.89	0.310	1.07	**0.549**	0.88
		y = 3.58x + 0.91		y = − 6.51x^2^ + 9.33x + 0.16		y = 1.33exp(1.18x)		y = −3.61exp(−0.08x) − 3.84exp(−4.21x)	
TRBI	[61]	**0.663**	0.75	0.639	0.75	0.625	0.79	0.659	0.76
		y = −2.55x + 3.62		y = 0.44x^2^ − 3.27x + 3.84		y = 4.39exp(−1.42x)		y = −7.13exp(−3.34x) − exp(−3.34x)	

**Table 5:** Best band combination for each generic vegetation index introduced in ARTMO from cross-validation of the testing dataset (VLC17_ES), ordered from highest to lowest R^2^, with a linear fit.

Index	Bands	R^2^	RMSE	NRMSE (%)	p-Value
TRBI	2190;740;865	0.737	0.63	14	<0.001
TDBI	2190;865;740	0.732	0.64	15	<0.001
ND3b	2190;865;740	0.731	0.64	15	<0.001
MNI	2190;865;1610	0.731	0.64	15	<0.001
RI	2190;865	0.728	0.64	15	<0.001
MCGI	1610;865;740	0.717	0.65	15	<0.001
TGI	1610;842;2190	0.713	0.66	15	<0.001
GLH	2190;1610;842	0.708	0.67	15	<0.001
NDGI	865;705	0.708	0.67	15	<0.001
MTGI	2190;865;490	0.701	0.68	15	<0.001

**Table 6:** Linear cross-validation fitting results of the normalized difference ratio index with different band combinations and LAI_green_ values from the testing and validation datasets, ordered from highest to lowest R^2^ according to the testing dataset.

Bands	Testing (VLC17_ES)		Validation (FOG17_IT)	
	R^2^	RMSE	R^2^	RMSE
865;705	0.708	0.67	0.732	0.69
783;705	0.702	0.68	0.711	0.71
842;705	0.688	0.69	0.717	0.71
740;705	0.685	0.71	0.686	0.74
783;665	0.675	0.71	0.678	0.75
842;665	0.665	0.72	0.684	0.74
783;740	0.531	0.85	0.674	0.76
